# Comparative Silk Transcriptomics Illuminates Distinctive Impact of Artificial Selection in Silkworm Modern Breeding

**DOI:** 10.3390/insects13121163

**Published:** 2022-12-16

**Authors:** Kesen Zhu, Yanfei Chen, Lei Chen, Hui Xiang

**Affiliations:** 1 Guangdong Provincial Key Laboratory of Insect Developmental Biology and Applied Technology, Guangzhou Key Laboratory of Insect Development Regulation and Application Research, School of Life Sciences, South China Normal University , Guangzhou 510631, China; 2 Laboratory for Lingnan Modern Agriculture, Institute of Insect Science and Technology, Guangzhou 510642, China; 3 Henry Fok School of Biology and Agriculture, Shaoguan University, Shaoguan 512000, China; 4 School of Ecology and Environment, Northwestern Polytechnical University, Xi’an 710072, China

**Keywords:** silkworm, improvement, silk gland, silk cocoon protein genes, nitrogen

## Abstract

**Simple Summary:**

To understand the molecular mechanism of the cocoon silk evolution of silkworms, we systematically compared the silk gland transcriptomes among the wild, early domestic, and improved silkworms and indicated that modern breeding in silkworms brought drastic expressional changes of genes compared with early domestication. Improved silkworms demonstrated repressed basic nitrogen synthesis metabolism, whereas they had enhanced dynamics of protein post-translation modification. In addition, we highlighted one candidate improvement gene up-regulated in the silk glands of the improved silkworms that is involved in the function of the nervous system.

**Abstract:**

Early domestication and the following improvement are two important processes in the cocoon silk evolution of silkworms. In contrast to early domestication, understanding of the improvement process is still fuzzy. By systematically comparing the larval silk gland transcriptomes of the wild, early domestic, and improved silkworms, we highlighted a novel landscape of transcriptome in the silk glands of improved ones. We first clarified that silk cocoon protein genes were up-regulated in modern breeding but not in early domestication. Furthermore, we found that differentially expressed genes (DEGs) between improved and early domestic silkworms (2711), as well as between improved and wild silkworms (2264), were obviously more than those between the early domestic and wild silkworms (158), with 1671 DEGs specific in the improved silkworm (IS-DEGs). Hierarchical clustering of all the DEGs consistently indicated that improved silkworms were significantly diverged from the early domestic and wild silkworms, suggesting that modern breeding might cause prompt and drastic dynamic changes of gene expression in the silk gland. We further paid attention to these 1671 IS-DEGs and were surprised to find that down-regulated genes were enriched in basic organonitrogen compound biosynthesis, RNA biosynthesis, and ribosome biogenesis processes, which are generally universally expressed, whereas those up-regulated genes were enriched in organonitrogen compound catabolic processes and functions involving in the dynamic regulation of protein post-translation of modification. We finally highlighted one candidate improvement gene among these up-regulated IS-DEGs, i.e., GDAP2, which may play roles in silk behavior and the overall robustness of the improved silkworm. The findings strongly suggest that modern breeding may facilitate effective control of the basic consumption of nitrogen and a stronger switch of nitrogen resources from other tissues to the silk glands, for an efficient supply for silk production, and implies the importance of brain behavior and robustness in silk yield improvement of modern breeding.

## 1. Introduction

As the only fully domesticated insect, the silkworm contributes to human beings with a great amount of silk. Cocoon silk is mainly composed of silk fiber proteins, i.e., fibroin heavy chain (Fib-H), fibroin light chain (Fib-L), and 25-kD polypeptide proteins (P25) [1], as well as sericins [2]. In the processes of silkworm domestication and improvement, cocoon silk yields have experienced two significant elevations. Although there are many efforts to explore the mechanism of silk gland development as well as silk synthesis [3,4,5,6], these studies could not address the improvement of silk gland and silk yield during silkworm evolution. Recent advances began to understand this issue from an evolutionary and omics view. The first resequencing analysis of 40 domestic (*Bombyx mori*) and wild (*B. mandarina*) silkworms proposed a batch of candidate domestication genes, among which were some transcription factors that regulate the expression of silk protein genes, suggesting that transcription factors may play a role in increasing cocoon silk quantity in silkworm domestication [7]. Our previous study, on a larger batch of silkworm resequencing, for the first time distinguished artificial selection on domestication and improvement, respectively, highlighting an efficient nitration utilization in both processes [8]. Recently, Tong et al. generated a comprehensive pan-genomic analysis of silkworms and illustrated genes affecting silk yield in silkworm improvement processes [9]. These efforts have been boosting our understanding of silkworm post-domestication evolution, especially on the improvement of silk economic traits.

Gene expressions in the silk gland, the silk-producing organ, have a great impact on silk’s economic traits. A few other studies have tried to explore the compact of artificial selection in this organ via silk gland comparative transcriptomic or proteomics analysis [10,11,12,13,14]. For example, Fang et al. found that up-regulated genes or pathways in the domestic silkworm were related to tissue development, protein secretion, and metabolism [10]. Zhou et al. generated a more comprehensive comparative analysis and suggested that the increase in silk yield during silkworm domestication was associated with the improvement of biological systems that included not only the expansion of silk gland cells but also a high expression of silk-coding genes and silk production-related genes [11]. Li [12] and Wang [14] respectively compared silk gland transcriptome and proteomes between high and low silk-yield silkworm strains. They consistently found that genes and pathways related to the biosynthesis of proteins were up-regulated in high silk yield strains [12,14]. Despite these findings on the mechanism of the enhancement of the cocoon silk yield during silkworm evolution, these efforts did not clearly distinguish the domestication and improvement process, which are two important steps in the domestication evolution [8,15]. Artificial selections in these two steps are different in multiple aspects, and their impacts on silk economic traits differ correspondingly [8,15].

In this study, we generated silk comparative transcriptomic analysis among the wild silkworm *B. mandarina*, and the local and improved groups of the domestic silkworm *B. mori*. We demonstrated a novel transcriptomic landscape in the silk glands of the improved silkworms and discovered candidate-improved genes with expression changes therein.

## 2. Materials and Methods

### 2.1. Sample Collection and RNA Extraction

Silk gland transcriptome data of four varieties from improved silkworms (*B. mori*) was generated in this study. The improved silkworm strains were provided by the School of Biotechnology, Jiangsu University of Science and Technology, Zhenjiang, China. Silk glands were dissected from the third day of the fifth instar silkworm larvae, reserved in dry ice, and sent to Novogene for further RNA extraction and RNA-seq. Briefly, Total RNA was extracted using TRIzol reagent (Invitrogen, Carlsbad, CA, USA) following the manufacturer’s instructions.

### 2.2. Data Collection

Silk gland transcriptome data of the (*B. mandarina*) (SRR6476552, SRR1592738, SRR6476548, SRR6476599) and four early domestic (*B. mori*) silkworms (SRR6476546, SRR6476594, SRR6476598, SRR6476551) were obtained from NCBI database (Accession: PRJNA428294 ID: 428294, https://www.ncbi.nlm.nih.gov/bioproject/PRJNA428294, accessed on 3 January 2018; Accession: PRJNA262539, https://www.ncbi.nlm.nih.gov/bioproject/PRJNA262539, accessed on 29 September 2014). The *B. mori* reference genome information (https://silkdb.bioinfotoolkits.net/base/download/-1, accessed on 5 January 2022) as well as spatial and temporal expression data of the coding genes (https://silkdb.bioinfotoolkits.net/__resource/Bombyx_mori/download/expression.txt, accessed on 5 January 2022) were obtained from SilkDB 3.0 database (https://silkdb.bioinfotoolkits.net/main/species-info/-1, accessed on 5 January 2022) [16]. The reference gene set information for artificial selection analysis and the genomic single nuclear polymorphic data (SNP) file (VCF) for the *B. mori* and *B. mandarina* were obtained from DEYAD platform (https://doi.org/10.5061/dryad.fn82qp6, accessed on 5 January 2022) [8].

### 2.3. RNA-Seq

mRNAs were enriched from total RNA by magnetic beads with Oligo (dT). The first-strand cDNA was synthesized with random hexamers using mRNA, which was cut into short fractions as the template, and the second-strand cDNA was synthesized when buffer, dNTPs, RNase H, and DNA polymerase I were added. The terminal repairing and adding poly A tail and the sequencing adaptor were performed after purification with a QiaQuick PCR kit and elution with EB buffer. The right-sized fragments were selected by agarose gel electrophoresis and amplified by PCR. The constructed library was sequenced by Illumina HiSeq™ 2000.

### 2.4. RNA-Seq Data Processing and SNP Calling

The clean RNA-seq data were mapped to the silkworm reference genome using STAR 2.7.9a [17] with default parameters. Bam files were sorted using SortSam and duplicated reads were marked with Picard v.2.21.4 (http://broadinstitute.github.io/picard/, accessed on 5 January 2022). Then, the SNPs were detected and filtered using HaplotypeCaller and VariantFiltration commands in GATK 4.0.12 (https://github.com/broadinstitute/gatk, accessed on 5 January 2022) [18]. To reduce the false discovery rate, the filtering steps followed these criteria: QD < 2.0, MQ < 40.0, FS > 60.0, SOR > 3.0, MQRankSum < −12.5, ReadPosRankSum < −8.0. We performed principal component analysis (PCA) with population-scale SNPs using the packages Plink [19], and the significance level of the eigenvectors was determined using the Tracy–Widom test.

### 2.5. Expression Quantification and Identification of Differential Expressed Genes

Expression quantification of each gene was calculated by salmon 0.11.3 [20] (https://github.com/COMBINE-lab/salmon, accessed on 5 January 2022) and shown as TPM (Transcripts Per Kilobase per Million mapped reads). As to identify DEGs, read counts of each gene were analyzed by R package Deseq2 1.32.0 [21] and were used for pair-wise differential expression analysis between every two groups of silkworm strains, using logFC ≥ 2 and FDR ≤ 0.05 as a filtering criterion.

### 2.6. GO and KEGG Enrichment Analysis

GO and KEGG enrichment analyses of candidate genes were performed using the online platform (https://www.omicshare.com/tools/Home/Soft/gogseasenior for GO enrichment and https://www.omicshare.com/tools/Home/Soft/pathwaygseasenior for KEGG enrichment respectively, accessed on 5 January 2022). Differential expressed genes were used as candidate groups for enrichment analysis, and all genes of the silkworm genome were used as background. Significance was determined by a hypergeometric test.

### 2.7. Analysis of the Artificial Signature

We checked the candidate improvement genes identified by our previous study [8] and identified 5 in our IS-DEGs by blast. Considering different gene ID in two studies, we used blastp 2.6.0 to establish the correspondence of two sets of gene IDs. Indexes (Fst and π) were used and plotting of the artificial signature, as well as determination of regions of signature, were the same as the ones in the above study [8].

## 3. Results

There was 6.5~11.1 Gb of clean data obtained for each of the 12 silkworm samples. The mapping rate ranged from 61.94% to 84.84% (Table S1). The wild silkworms showed a slightly lower mapping rate than the domestic ones due to genetic divergence between the two species. We characterized the genetic relationships among all sequenced strains using PCA, which was consistent with the previous research results. The results confirmed that the improved and the early domesticated silkworm strains were genetically divergent from wild silkworms (Figure S1).

### 3.1. Silk Cocoon Protein Genes Were Obviously More Highly Expressed in the Silk Gland of Improved Silkworm Strains

**Figure 1:**
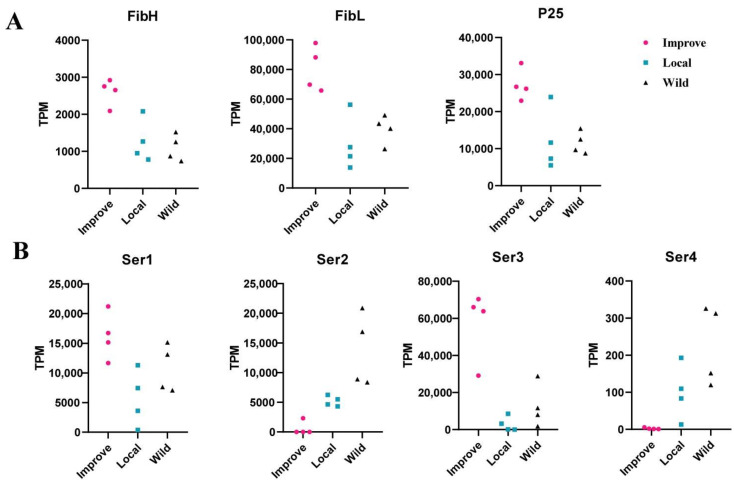
Expression of the silk-coding genes in different silkworm groups. Improve, the improved silkworm (*Bombyx mori*) strains; Local, the early domesticated silkworm (*B. mori*) strains; Wild, the wild silkworm (*B. mandarina*) different geographical individuals. (**A**) Expression of silk fiber protein genes. (**B**) Expression of the for the sericin genes.

We investigated the expression of the silk fibroin genes, i.e., *FibH*, *FibL*, and *P25*, as well as the sericin genes. Interestingly, we found that there was no significant difference between the early domestic and wild silkworms in terms of all the silk fibroin genes, but a drastic elevated expression of them occurred in the improvement silkworms (Figure 1A), suggesting that the increase of the silk yield may not be so important during the early domestication process, whereas it may be the key target in modern breeding. A similar pattern was also observed in *Ser1* and *Ser3*, two sericin genes that were reported to be components of the cocoon [22]. Ser2 and Ser4, which were not in the cocoon silk [2,22], showed extremely low expression in the improved silkworm, reflecting the favorite choice for the efficient control of the allocation of nitrogen resources in modern breeding (Figure 1B).

### 3.2. Improved Silkworms Showed Specific Expression Patterns in the Silk Gland Compared with the Early Domestic and Wild Silkworms

**Figure 2:**
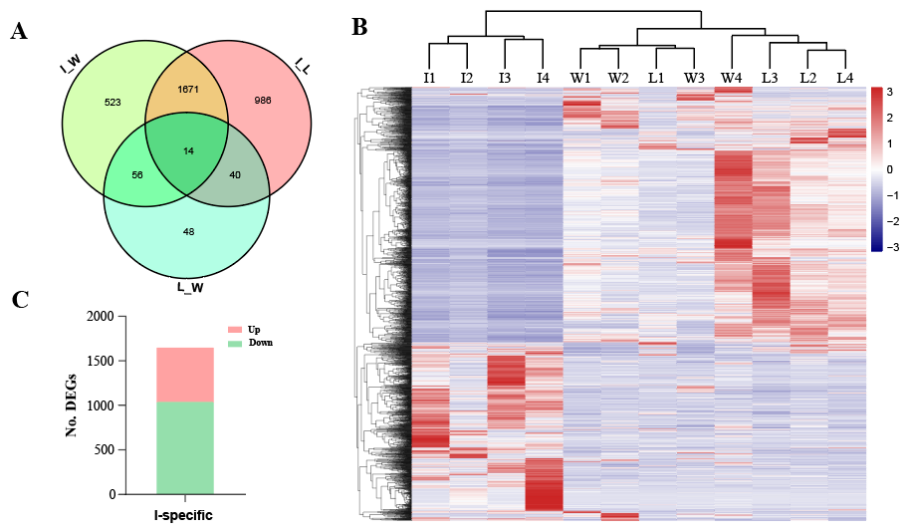
Differentially expressed genes (DEGs) in the silk gland among the wild, the early domestic and improved silkworms. (**A**) Venn scheme of the pairwise differentially expressed genes among different silkworm groups; (**B**) heatmap and hierarchical cluster of all the DESs among different silkworm groups. I, the improved silkworm (*B. mori*) strains; L, the early domestic silkworm (*B. mori*) strains; Wild, the different wild silkworm (*B. mandarina*) geographical individuals. (**C**) Information of the improved silkworm-specific differentially expressed genes (IS-DEGs). Up, up-regulated; Down, down-regulated.

In total, we identified 2711 DEGs between the improved (I) and early domesticated (L) silkworm (*B. mori*) strains (I vs. L DEGs) and 2264 DEGs between the improved (*B. mori*) and wild (*B. mandarina*) (W) silkworms (I vs. W DEGs), with 1671 DEGs specific in the improved silkworm (Figure 2A) (IS-DEGs). The two sets of improved silkworm-related DEGs (i.e., 2711 and 2264) were drastically more than those between the early domestic and wild silkworms (158 L vs. W DEGs) (Figure 2A). The results suggested that the improved silkworm strains might be distinctive in silk gland gene expression patterns from those in early domestic and wild silkworms. Consistently, hierarchical clustering of silk gland DEGs indicated that the early domestic silkworms and the wild silkworms were clustered in one group, whereas the improved silkworm strains were in the other (Figure 2B). This result was interesting since we observed an obviously intra-specific (*B. mori* I vs. L groups) difference, compared with the inter-specific (*B. mori* vs. *B. mandarina*) difference, in terms of silk gland expression pattern. We speculate that modern breeding of silkworm for the rapid improvement of silk yield has had a more intensive compact on silk glands compared with the early domestication process, in which adaptation for domestic conditions of the wild silkworms might be crucial.

**Figure 3:**
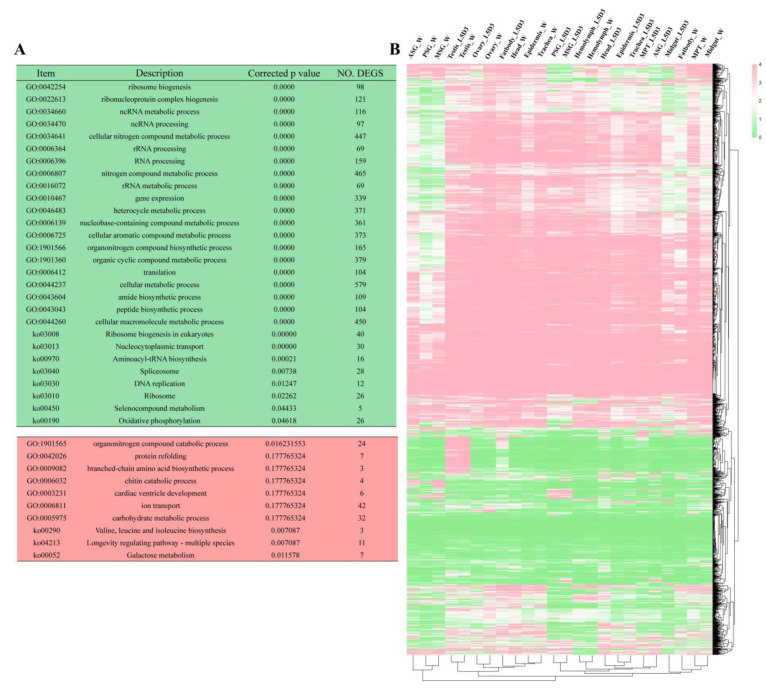
Functional enchainment and the tissue expression pattern of the 1671 improved silkworm-specific DEGs (IS-DEGs). (**A**) Functional enrichments of up-regulated (pink) and down-regulated IS-DEGs (green). (**B**) Tissue expression pattern of the IS-DEGs in the enriched biological processes and pathways in the local strain (P50) of the domestic silkworm. MPT, Malpighian tubule; ASG\MSG\PSG, anterior\Middle\Posterior silk gland; L5D3, the 3rd day of the 5th instar larval stage; W, the wandering stage.

We further paid attention to the 1671 IS-DEGs (Table S2). Considering that the early domestic and wild silkworms were similar in silk gland transcriptomic pattern and these 1671 genes were not differentially expressed between them (Figure 2A), we supposed that they might play an important role in silk gland improvement during the modern breeding process. Among them, down-regulated DEGs were significantly more than the up-regulated ones (1047 vs. 624, *p* < 0.001, by Chi-square test) (Figure 2C). These down-regulated DEGs were significantly enriched in such biological processes as an organonitrogen compound biosynthetic process, RNA biosynthesis, and ribosome biogenesis processes (Figure 3A). They were consistently enriched by pathways such as the ribosome, ribosome biogenesis in eukaryotes, and amino-acyl-tRNA biosynthesis (Figure 3A). These biological processes or pathways were related to the biosynthetic process of proteins. To our surprise, they were distinctly repressed in the silk gland of the improved silkworm strains, where silk protein synthesis was being synthesized abundantly (Figure 1).

Contrarily, up-regulated DEGs were enriched in organonitrogen compound catabolic process with substantial significance (corrected *p*-value < 0.05), and other processes such as protein refolding, chitin catabolic process, branched-chain amino acid biosynthetic processes, ion transport and carbohydrate metabolic process with marginal significance (Figure 3A). In terms of KEGG, they were significantly enriched in such pathways as valine, leucine, and isoleucine biosynthesis, longevity regulating pathway, and galactose metabolism (Figure 3A). In the galactose metabolism pathway, there was one gene (alpha-N-acetylgalactosaminidase precursor) bearing the alpha-galactosidase [EC: 3.2.1.22] and functions in many steps (Figure S2).

### 3.3. Up- and Down-Regulated IS-DEGs in the above Enriched Biological Processes and Pathways Showed Different Tissue Expression Patterns

We further tested the tissue expression pattern of the IS-DEGs in the above enriched biological processes and pathways using the published silkworm transcriptome data (https://silkdb.bioinfotoolkits.net, accessed on 5 January 2022). Intriguingly, the expression heat map showed two sharply diverged clusters (Figure 3B). In the upper cluster, where almost all DEGs were down-regulated in the improved silkworm, most genes were ubiquitously and highly expressed in multiple tissues. Moreover, the majority of these genes showed slightly lower expression in the silk gland at the wandering stage (Figure 3B). On the contrary, few genes in the lower cluster were ubiquitously expressed and nearly all of these genes were up-regulated in the improved silkworm. At the wandering stage and in the silk gland, most of these genes showed substantial expression (Figure 3B). These results suggested that in the improved silkworm, the basic nitrogen-related metabolisms were repressed, but the transport process and active protein progress were promoted, maybe for the efficiency of silk protein synthesis and silk spinning.

### 3.4. Five Candidate Improvement Genes Showed Specific Expression Patterns in the Improved Silkworm Strains

**Figure 4:**
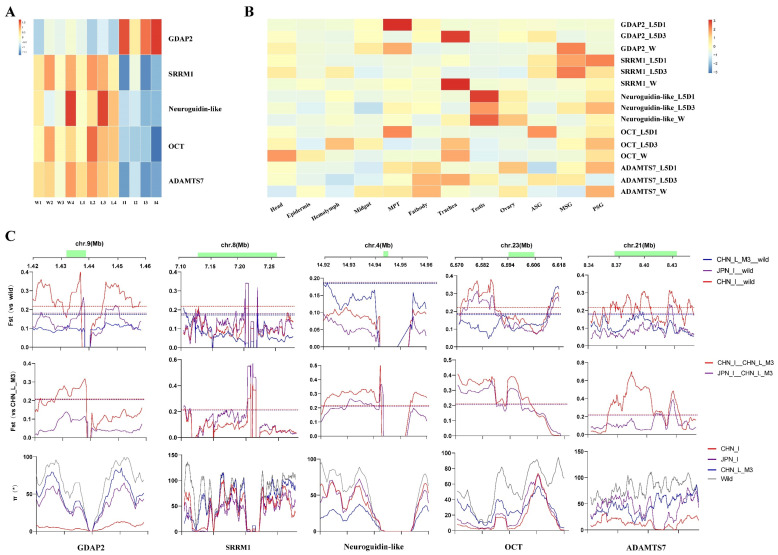
Expression and selection signature of the five candidate improvement genes. (**A**) Heat map of the expression in the silk glands of the five candidate improvement genes in different silkworm strains or individuals. I, the improved silkworm (*B. mori*) strains; L, the early domestic silkworm (*B. mori*) strains; Wild, the different wild silkworm (*B. mandarina*) geographical individuals. (**B**) Heat map of the tissue expression of the five candidate improvement genes in the local strain (P50) domestic silkworm. MPT, Malpighian tubule; ASG\MSG\PSG, anterior\Middle\Posterior silk gland respectively; L5D1/L5D3, the 1st/3rd day of the 5th instar larval stage; W, wandering stage. (**C**) Selection signatures for the five candidate improvement genes. The Fst plottings of each silkworm groups against wild silkworms (the **top panel**) and early domestic silkworm strains (the **mid panel**), as well as the π (the **bottom panel**) plotting are shown, along the genomic regions covering the five genes. Dashed lines represent the top 1% of values, respectively.

Among the 1671 IS-DEGs, we identified five candidate improvement genes [8] (Table S3). Four genes, i.e., *SRRM1*, *OCT*, *Neuroguidin-like*, and *ADAMTS7* were down-regulated DEGs except for *GDAP2*, which was up-regulated (Figure 4A). Temporal and spatial expression patterns of these genes in the silkworm local strain *P50* indicated that the *GDAP2* gene posterior silk gland (PSG) was lower expressed, whereas the other genes were PSG- higher expressed (Figure 4B), implying that modern breeding seemly enhanced expression of this otherwise relatively weak expressed PSG gene in the local silkworms.

We further checked the artificial selection signature of the five candidate improvement genes according to our previous approach [8] and excluded *Neuroguidin-like* and SRRM1, which showed no or weak artificial selection signatures in their genic region (Figure 4C). As to *OTC* (organic cation transporter protein isoform X1), the selective sweep locates in the upstream region in both Chinese and Japanese improved groups (Figure 4C). *GDAP2* showed a strong selection signature in the Chinese improvement silkworm group. Similarly, a slightly weak signature was observed in *ADAMTS7* (A disintegrin and metalloproteinase with thrombospondin motifs 7).

## 4. Discussion

Modern breeding is usually much more human-purposive and highly efficient [8,15]. As to the silkworm, increment of silk yield and silk production efficiency is the most important goal in the breeding process, compared to the early domestication process. Here in this study, comparative silk gland transcriptomic analysis among the wild, the early domestic, and the improved silkworms provides substantial supporting evidence that the modern breeding process would have much more impact in the silk-producing tissue, i.e., the silk gland, than the early domestication process. We clarified the differential expression pattern of cocoon silk-coding genes in the two evolutionary processes of silkworms (Figure 1), highlighting that substantially increased expression turns out to be in the silkworm improvement process, in contrast to previously rough documentation as up-regulation [11]. Moreover, the transcriptome landscape in the silk gland of the improved silkworm group is divergent from the wild and the early domestic silkworm groups (Figure 2). Consistently, much more DEGs were found in the improved silkworm group, compared with those between early domestic and wild silkworms (Figure 2). It is interesting since this pattern of silk gland gene expression is obviously distinguished from what the genome data usually demonstrates. At the genetic level, both early domestic and improved silkworms have sharply diverged from the wild silkworm [8,9]. We suspect that artificial selection in intensive modern breeding could cause prompt and drastic dynamic changes of gene expression in the silk-producing tissue, i.e., the silk gland, resulting in the prompt and drastic improvement of silk economic traits. Although, future large batches of sampling, covering the classical silkworm local and improved groups from different geographical origins, showing different silk cocoon-related characters, is needed for further illumination of this pattern. Notably, it is known that during the silkworm’s early domestication process, there was a substantial increase in silk yield. From the expression of silk protein genes, we could learn that, possibly, an increase in silk yield in this process may not be attributed to increased transcription of silk genes. It may benefit from better utilization of nitrogen. Gene expression in other tissues, such as hemolymph, fat body, and midgut, might be helpful in further in-depth exploration.

Our in-depth analyses on the improvement silkworm specific DEGs (IS-DEGs) illuminate interesting insights. Intriguingly, the results implied the overall repression of nitrogen biosynthesis and ribosome biogenesis in the silk gland, specifically in the modern breeding process, which was remarkably different from previous reports that were documented as up-regulation [11,12,23]. This novel pattern is supported by our recent study on silkworm heterosis [24], in which we found that one alternative mechanism may be the down-regulation of pathways involving basic nitrogen synthesis. We hence supposed that rapid improvement of silk yield could benefit from the repression of nonspecific consumption of nitrogen. We further tested the tissue expression pattern of genes in these pathways or biological processed and found that most of them were universally expressed (Figure 3), with slightly lower expression in the posterior silk glands of the local silkworm strain, further supporting our conjecture. Silk protein synthesis is a tightly regulated process affected by many factors. From the view of nutrition and metabolism, when the silkworm larvae entered the last instar, the retention rate of mulberry leaf protein in silkworm tissues outside silk glands decreased from 64% to 4%. However, in the silk glands, the amount of protein transferred from other tissues and organs of the silkworm gradually increased from 9% to 96% [25]. Before the spinning process, the silkworm has a strong switch of the nitrogen resources from other tissues to the silk glands, in order to support the necessity for silk production. Modern breeding might prefer higher efficiency of this kind of switch by repressing the basic protein and nitrogen metabolism of the other tissues and instead promoting reallocation and transport system of nitrogen resources to the silk gland. Furthermore, consistent with this hypothesis, we found that up-regulated IS-DEGs were mainly enriched in biological processes and functions related to the organonitrogen compound catabolic process (Figure 3).

Notably, we found some other enriched biological processes and pathways of the up-regulated IS-DEGs (Figure 3), providing new cues in modern breeding. Firstly, the enhanced organonitrogen compound catabolic process suggested efficient protein degradation. Secondly, in the galactose metabolism pathway, which belonged to the carbohydrate metabolic process, we noticed the silkworm N-acetylgalactosaminidase (GalNAcase) gene, which removed the O-linked α-N-acetylgalactosamine (GalNAc) residue from a glycopeptide, for protein degradation [26]. In contrast, another type of enzyme, i.e., N-acetylgalactosaminyltransferase (GalNAcT), transfers GalNAc to a specific residue of a polypeptide in the Golgi apparatus [27]. The two types of enzymes both play roles in the dynamic regulation of protein glycosylation, which is an important post-translational protein modification in the silk gland [28,29]. Factually, there are diverse members of the two enzyme types in insects [26,29]. Here, we totally identified five genes bearing GalNAcase (1) or GalNAcT (4) activity in all of the IS-DEGs, with three up-regulated (one GalNAcase; two GalNAcTs) and two (GalNAcTs) down-regulated (Figure S3). Ile/Leu residue of the GalNAcT is reported to be a key determinant affecting donor sugar specificity [30]. Consistently, we observed the enriched pathways, valine, leucine, and isoleucine biosynthesis pathway were consistent with the branched-chain amino acid biosynthetic process. Based on the above analyses, we suspect that in the silk glands of the improved silkworm, active bidirectional regulation on protein glycosylation may occur, which benefits the improved silkworm’s better capacity in the re-use of nitrogen resources as well as effective protein post-translation modification, for efficient silk production.

Artificial selection during modern silkworm breeding for an increased silk yield might not restrict to direct utilization of nitrogen resources [8]. Genes or pathways related to the neural system may be also helpful [24,31,32]. Here, we highlighted a candidate improved gene, i.e., *GDAP2,* which encodes ganglioside-induced differentiation-related protein 2, and was an up-regulated IS-DEGs in the silk gland (Figure 4). *GDAP2* has complex functions in signal transduction, transportation, and organelle biology, and might be involved in cellular metabolic or stress responses [33]. In humans, *GDAP2* is highly expressed in the brain [34]. When *GDAP2* was up-regulated, the complexity of neurons and the number of dendritic spines increased [34]. In *Drosophila*, ubiquitous knockdown of *GDAP2* resulted in a shortened lifespan and motor behavior anomalies such as righting defects, reduced and uncoordinated walking behavior, and compromised flight. *GDAP2* knockdown flies showed increased sensitivity to deleterious effects of stressors such as reactive oxygen species and nutrient deprivation [33]. In the silkworm, silk spinning is also a high motor behavior, especially of the head, and may involve complicated brain activity [35]. Therefore, increased silk yield may also be due to the improved silkworm being overall more robust. *GDAP2* may play a similar role in the silkworm’s enhanced behavior and tolerance to stresses. In general, up-regulated genes or pathways in the silk gland may also reflect imprints of modern breeding upon these superior characteristics of the improved silkworm as a whole.

## Data Availability

RNA-seq data of the four improved silkworms were deposited to the NCBI SRA database under the Accession SRR22571454, SRR22571455, SRR22571456, and SRR22571457.

## Supplementary Materials

The following are available online at https://www.mdpi.com/article/10.3390/insects13121163/s1, Figure S1: The PCA map constructed based on transcriptome data of all sequenced strains. Improve, the improved silkworm (*Bombyx mori*) strains; Local, the early domesticated silkworm (*B. mori*) strains; Wild, the wild silkworm (*B. mandarina*) different geographical individuals, Figure S2: The galactose metabolism pathway. The green box indicates that the gene (al-pha-N-acetylgalactosaminidase precursor) bearing the alpha-galactosidase [EC: 3.2.1.22] may participate in the reaction, Figure S3: Expression of three up-regulated (one GalNAcase; two GalNAcTs) and two (GalNAcTs) down-regulated genes in different silkworm groups. Improve, the im-proved silkworm (*Bombyx mori*) strains; Local, the early domesticated silkworm (*B. mori*) strains; Wild, the wild silkworm (*B. mandarina*) different geographical individuals. Table S1: Statistics of RNA-seq data and mapping rate, Table S2: Information of the 1671 IS-DEGs, Table S3: Positive selection genes differentially expressed in improved silkworm strains.

## References

[1] Inoue S., Tanaka K., Arisaka F., Kimura S., Ohtomo K., Mizuno S. (2000). Silk fibroin of Bombyx mori is secreted, assembling a high molecular mass elementary unit consisting of H-chain, L-chain, and P25, with a 6:6:1 molar ratio. J. Biol. Chem..

[2] Dong Z., Guo K., Zhang X., Zhang T., Zhang Y., Ma S., Chang H., Tang M., An L., Xia Q. (2019). Identification of Bombyx mori sericin 4 protein as a new biological adhesive. Int. J. Biol. Macromol..

[3] Hou S., Sun Y., Wu Y., Cheng T., Liu C. (2019). Bmsage is involved in the determination of cell number in the silk gland of Bombyx mori. Insect Biochem. Mol. Biol..

[4] Hu W., Chen Y., Lin Y., Xia Q. (2019). Developmental and transcriptomic features characterize defects of silk gland growth and silk production in silkworm naked pupa mutant. Insect Biochem. Mol. Biol..

[5] Shi R., Ma S., He T., Peng J., Zhang T., Chen X., Wang X., Chang J., Xia Q., Zhao P. (2019). Deep Insight into the Transcriptome of the Single Silk Gland of Bombyx mori. Int. J. Mol. Sci..

[6] Tang X., Liu H., Shi Z., Chen Q., Kang X., Wang Y., Zhao P. (2020). Enhanced silk yield in transgenic silkworm (*Bombyx mori*) via ectopic expression of BmGT1-L in the posterior silk gland. Insect Mol. Biol..

[7] Xia Q., Guo Y., Zhang Z., Li D., Xuan Z., Li Z., Dai F., Li Y., Cheng D., Li R. (2009). Complete resequencing of 40 genomes reveals domestication events and genes in silkworm (Bombyx). Science.

[8] Xiang H., Liu X., Li M., Zhu Y., Wang L., Cui Y., Liu L., Fang G., Qian H., Xu A. (2018). The evolutionary road from wild moth to domestic silkworm. Nat. Ecol. Evol..

[9] Tong X., Han M.J., Lu K., Tai S., Liang S., Liu Y., Hu H., Shen J., Long A., Zhan C. (2022). High-resolution silkworm pan-genome provides genetic insights into artificial selection and ecological adaptation. Nat. Commun..

[10] Fang S.M., Hu B.L., Zhou Q.Z., Yu Q.Y., Zhang Z. (2015). Comparative analysis of the silk gland transcriptomes between the domestic and wild silkworms. BMC Genom..

[11] Zhou Q.Z., Fu P., Li S.S., Zhang C.J., Yu Q.Y., Qiu C.Z., Zhang H.B., Zhang Z. (2020). A Comparison of Co-expression Networks in Silk Gland Reveals the Causes of Silk Yield Increase During Silkworm Domestication. Front. Genet..

[12] Li J., Qin S., Yu H., Zhang J., Liu N., Yu Y., Hou C., Li M. (2016). Comparative Transcriptome Analysis Reveals Different Silk Yields of Two Silkworm Strains. PLoS ONE.

[13] Wang S., You Z., Feng M., Che J., Zhang Y., Qian Q., Komatsu S., Zhong B. (2016). Analyses of the Molecular Mechanisms Associated with Silk Production in Silkworm by iTRAQ-Based Proteomics and RNA-Sequencing-Based Transcriptomics. J. Proteome Res..

[14] Wang S.H., You Z.Y., Ye L.P., Che J., Qian Q., Nanjo Y., Komatsu S., Zhong B.X. (2014). Quantitative proteomic and transcriptomic analyses of molecular mechanisms associated with low silk production in silkworm Bombyx mori. J. Proteome Res..

[15] Meyer R.S., Purugganan M.D. (2013). Evolution of crop species: Genetics of domestication and diversification. Nat. Rev. Genet..

[16] Lu F., Wei Z., Luo Y., Guo H., Zhang G., Xia Q., Wang Y. (2020). SilkDB 3.0: Visualizing and exploring multiple levels of data for silkworm. Nucleic Acids Res..

[17] Dobin A., Gingeras T.R. (2015). Mapping RNA-seq Reads with STAR. Curr. Protoc. Bioinform..

[18] McKenna A., Hanna M., Banks E., Sivachenko A., Cibulskis K., Kernytsky A., Garimella K., Altshuler D., Gabriel S., Daly M. (2010). The Genome Analysis Toolkit: A MapReduce framework for analyzing next-generation DNA sequencing data. Genome Res..

[19] Chang C.C., Chow C.C., Tellier L.C., Vattikuti S., Purcell S.M., Lee J.J. (2015). Second-generation PLINK: Rising to the challenge of larger and richer datasets. Gigascience.

[20] Patro R., Duggal G., Love M.I., Irizarry R.A., Kingsford C. (2017). Salmon provides fast and bias-aware quantification of transcript expression. Nat. Methods.

[21] Love M.I., Huber W., Anders S. (2014). Moderated estimation of fold change and dispersion for RNA-seq data with DESeq2. Genome Biol..

[22] Dong Y., Dai F., Ren Y., Liu H., Chen L., Yang P., Liu Y., Li X., Wang W., Xiang H. (2015). Comparative transcriptome analyses on silk glands of six silkmoths imply the genetic basis of silk structure and coloration. BMC Genom..

[23] Guo Y., Shen Y.H., Sun W., Kishino H., Xiang Z.H., Zhang Z. (2011). Nucleotide diversity and selection signature in the domesticated silkworm, Bombyx mori, and wild silkworm, Bombyx mandarina. J. Insect Sci..

[24] Xu H., Chen L., Tong X.L., Hu H., Liu L.Y., Liu G.C., Zhu Y.N., Zhao R.P., Wang W., Dai F.Y. (2022). Comprehensive silk gland multi-omics comparison illuminates two alternative mechanisms in silkworm heterosis. Zool. Res..

[25] Li Q.Y., Xun L.J., Liao P.F. (2016). Main Factors Influencing Silk Production of Silkworm. Shandong Agric. Sci..

[26] Ikegaya M., Miyazaki T., Park E.Y. (2021). Biochemical characterization of Bombyx mori alpha-N-acetylgalactosaminidase belonging to the glycoside hydrolase family 31. Insect Mol. Biol..

[27] Xu J., Morio A., Morokuma D., Nagata Y., Hino M., Masuda A., Li Z., Mon H., Kusakabe T., Lee J.M. (2018). A functional polypeptide N-acetylgalactosaminyltransferase (PGANT) initiates O-glycosylation in cultured silkworm BmN4 cells. Appl. Microbiol. Biotechnol..

[28] Kajiura H., Eguchi T., Uchino K., Tatematsu K.I., Tamura T., Sezutsu H., Fujiyama K. (2022). Temporal analysis of N-acetylglucosamine extension of N-glycans in the middle silk gland of silkworm Bombyx mori. J. Biosci. Bioeng..

[29] Kajiura H., Miyauchi R., Kakudo A., Ohashi T., Misaki R., Fujiyama K. (2021). Bombyx mori beta1,4-N-acetylgalactosaminyltransferase possesses relaxed donor substrate specificity in N-glycan synthesis. Sci. Rep..

[30] Ramakrishnan B., Qasba P.K. (2007). Role of a single amino acid in the evolution of glycans of invertebrates and vertebrates. J. Mol. Biol..

[31] Li C., Zuo W., Tong X., Han M., Gao R., Hu H., Lu K., Luan Y., Zhang B., Liu Y. (2021). Whole-genome resequencing reveals loci under selection during silkworm improvement. J. Anim. Breed. Genet..

[32] Cui Y., Liu Z.L., Li C.C., Wei X.M., Lin Y.J., You L., Zhu Z.D., Deng H.M., Feng Q.L., Huang Y.P. (2021). Role of juvenile hormone receptor Methoprene-tolerant 1 in silkworm larval brain development and domestication. Zool. Res..

[33] Eidhof I., Baets J., Kamsteeg E.J., Deconinck T., van Ninhuijs L., Martin J.J., Schule R., Zuchner S., De Jonghe P., Schenck A. (2018). GDAP2 mutations implicate susceptibility to cellular stress in a new form of cerebellar ataxia. Brain.

[34] Hu D., Guo Y., Wu M., Ma Y., Jing W. (2022). GDAP2 Overexpression Affects the Development of Neurons and Dysregulates Neuronal Excitatory Synaptic Transmission. Neuroscience.

[35] Li Y., Wang X., Chen Q., Hou Y., Xia Q., Zhao P. (2016). Metabolomics Analysis of the Larval Head of the Silkworm, Bombyx mori. Int. J. Mol. Sci..

