# Supplementary material for: Comparative Silk Transcriptomics Illuminates Distinctive Impact of Artificial Selection in Silkworm Modern Breeding
Source: Insects. 2022 Dec 16;13(12):1163. doi: 10.3390/insects13121163 (PMC9784016; doi:10.3390/insects13121163)
Supplement: Supplementary file 1 [file insects-13-01163-s001.zip › Fig S1.pdf]

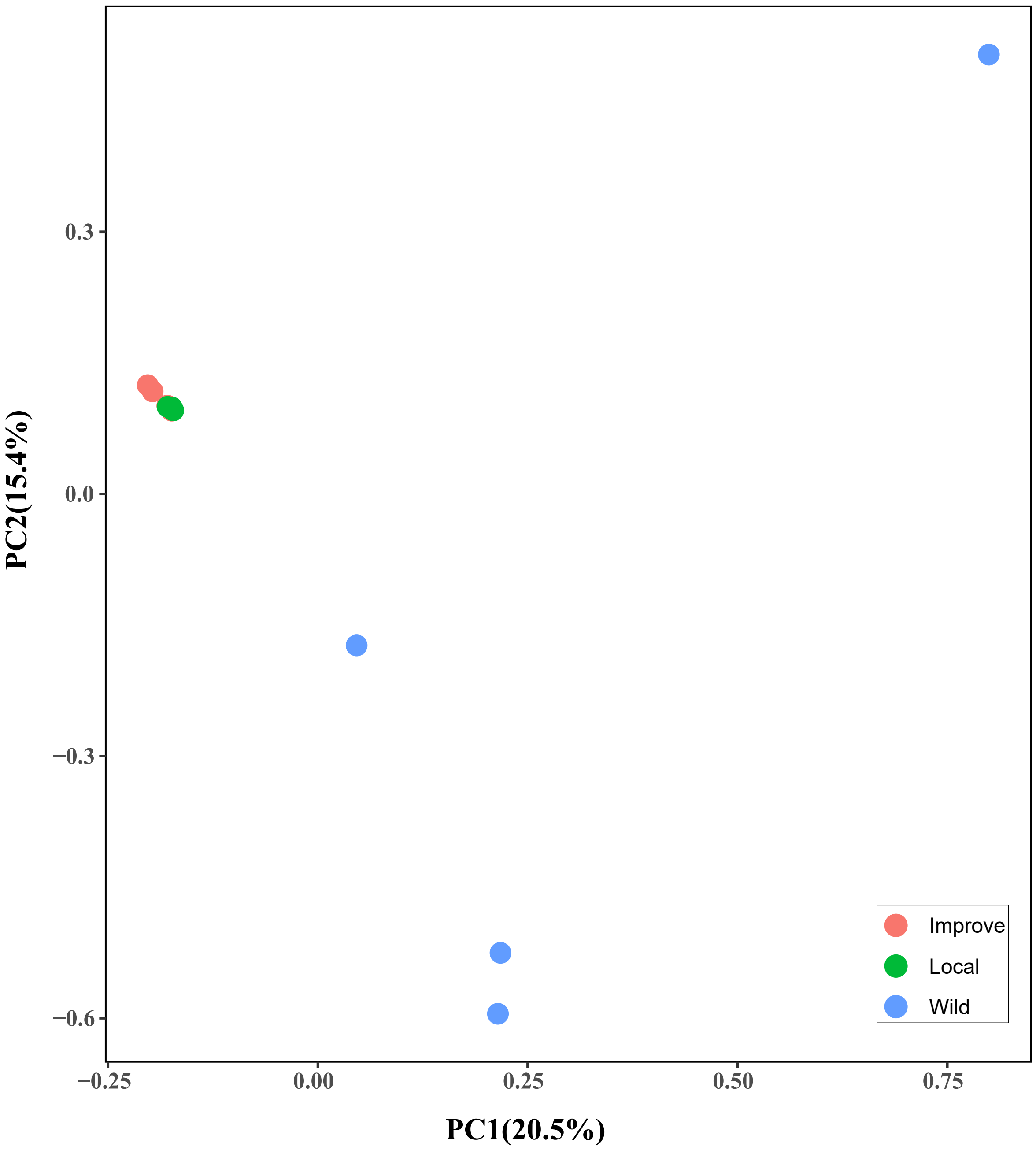

Figure S1. The PCA map constructed based on transcriptome data of all sequenced strains. Improve, the improved silkworm (*Bombyx mori*) strains; Local, the early domesticated silkworm (*B. mori*) strains; Wild, the wild silkworm (*B. mandarina*) different geographical individuals.
