# Supplementary material for: Comparative Silk Transcriptomics Illuminates Distinctive Impact of Artificial Selection in Silkworm Modern Breeding
Source: Insects. 2022 Dec 16;13(12):1163. doi: 10.3390/insects13121163 (PMC9784016; doi:10.3390/insects13121163)
Supplement: Supplementary file 1 [file insects-13-01163-s001.zip › Fig S2.pdf]

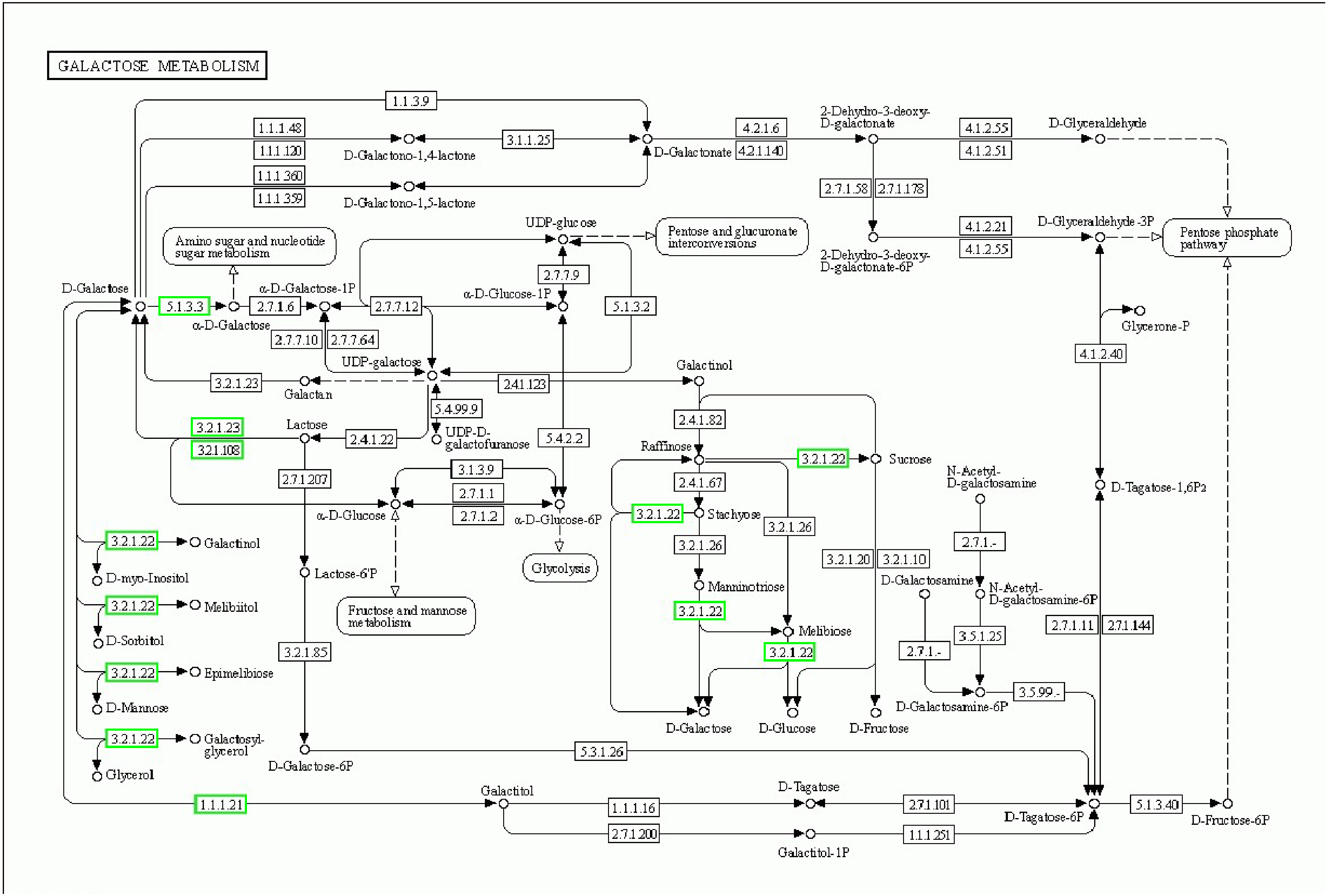

Figure S2. The galactose metabolism pathway. The green box indicates that the gene (al-pha-N-acetylgalactosaminidase precursor) bearing the alpha-galactosidase [EC: 3.2.1.22] may participate in the reaction.
