# Supplementary material for: Comparative Silk Transcriptomics Illuminates Distinctive Impact of Artificial Selection in Silkworm Modern Breeding
Source: Insects. 2022 Dec 16;13(12):1163. doi: 10.3390/insects13121163 (PMC9784016; doi:10.3390/insects13121163)
Supplement: Supplementary file 1 [file insects-13-01163-s001.zip › Supplementary Table 1.pdf]

Supplementary Table 1: Statistics of RNA-seq data and mapping rate

ID	Clean_reads	Clean_data(bp)	Mapping rate	Accession Number
**W1**	**31,442,000**	**10,331,415,588**	**70.96%**	SRR6476552
**W2**	**31,710,704**	**11,107,735,274**	**64.32%**	SRR1592738
**W3**	**28,534,127**	**9,371,817,498**	**73.44%**	SRR6476548
**W4**	**24,894,384**	**8,170,702,308**	**73.91%**	SRR6476599
**L1**	**26,867,168**	**8,821,721,028**	**77.35%**	SRR6476546
**L2**	**29,706,661**	**9,758,753,718**	**84.84%**	SRR6476594
**L3**	**30,202,803**	**9,922,480,578**	**87.17%**	SRR6476598
**L4**	**24,789,438**	**8,136,070,128**	**84.86%**	SRR6476551
**I1**	**21,692,839**	**6,583,482,042**	**70.14%**	SRR22571455*
**I2**	**29,739,625**	**9,025,572,505**	**75.93%**	SRR22571456*
**I3**	**24,592,537**	**7,463,507,771**	**61.94%**	SRR22571457*
**I4**	**25,016,234**	**7,592,079,339**	**70.24%**	SRR22571454*

*data were generated by this study.
