# Supplementary material for: Comparative Silk Transcriptomics Illuminates Distinctive Impact of Artificial Selection in Silkworm Modern Breeding
Source: Insects. 2022 Dec 16;13(12):1163. doi: 10.3390/insects13121163 (PMC9784016; doi:10.3390/insects13121163)
Supplement: Supplementary file 1 [file insects-13-01163-s001.zip › Supplementary Table 3.pdf]

Supplementary Table 3: Positive selection genes differentially expressed in improved silkworm strains

Bmk ID	Gene ID	Gene annotation
BMSK0004807.1	Bmor_03280	Protein GDAP2 homolog, partial
BMSK0004399.1	Bmor_04795	Serine/arginine repetitive matrix protein 1
BMSK0002082.1	Bmor_05550	Neuroguidin-like
BMSK0013210.1	Bmor_07031	Organic cation transporter protein isoform X1
BMSK0012180.1	Bmor_12065	A disintegrin and metalloproteinase with thrombospondin motifs 7
